# Supplementary material for: Global research on syndemics: a meta-knowledge analysis (2001-2020)
Source: F1000Res. 2023 Feb 2;11:253. Originally published 2022 Mar 1. [Version 2] doi: 10.12688/f1000research.74190.2 (PMC10015119; doi:10.12688/f1000research.74190.2)
Supplement: Peer review history

# Peer review: Reviewer response for version 2

**DOI:** 10.5256/f1000research.143637.r162173

**Published:** 2023-03-14

**Authors:** Ashley V. Hill

**Affiliations:** 1 Department of Epidemiology, University of Pittsburgh School of Public Health, Pittsburgh, PA, USA

I have reviewed the revisions to this manuscript and am approving for indexing.

Is the work clearly and accurately presented and does it cite the current literature?

Partly

If applicable, is the statistical analysis and its interpretation appropriate?

I cannot comment. A qualified statistician is required.

Are all the source data underlying the results available to ensure full reproducibility?

Yes

Is the study design appropriate and is the work technically sound?

Yes

Are the conclusions drawn adequately supported by the results?

Partly

Are sufficient details of methods and analysis provided to allow replication by others?

Yes

Reviewer Expertise:

NA

I confirm that I have read this submission and believe that I have an appropriate level of expertise to confirm that it is of an acceptable scientific standard.

# Peer review: Reviewer response for version 2

**DOI:** 10.5256/f1000research.143637.r162172

**Published:** 2023-02-13

**Authors:** Nicola Bulled

**Affiliations:** 1 InCHIP, University of Connecticut, Storrs, CT, USA; 2 School of Public Health, Brown University, Providence, RI, USA

A sound and thoughtful review of the literature, offering valuable insights on the development and application of this important and useful theoretical perspective.

Is the work clearly and accurately presented and does it cite the current literature?

Partly

If applicable, is the statistical analysis and its interpretation appropriate?

Yes

Are all the source data underlying the results available to ensure full reproducibility?

Yes

Is the study design appropriate and is the work technically sound?

Yes

Are the conclusions drawn adequately supported by the results?

Partly

Are sufficient details of methods and analysis provided to allow replication by others?

Yes

Reviewer Expertise:

NA

I confirm that I have read this submission and believe that I have an appropriate level of expertise to confirm that it is of an acceptable scientific standard.

# Peer review: Reviewer response for version 1

**DOI:** 10.5256/f1000research.77914.r125794

**Published:** 2022-03-29

**Authors:** Ashley V. Hill

**Affiliations:** 1 Department of Epidemiology, University of Pittsburgh School of Public Health, Pittsburgh, PA, USA

The authors of the present article provided a robust review of the literature examining “Syndemics” in multiple areas of emphasis and with various health indicators and outcomes. This article attempts to provide an overview of syndemic named literature published in the past 20 years and highlights both the researchers, institutions, and countries within which this work is being conducted.

 An understanding of the scope of syndemic work being conducted globally is helpful, however, the article lacks any examination of the statistical approaches used across the articles included and overlooks the relatively robust discourse in the literature on criticisms of commonly used analytical approaches to syndemic research. As mentioned by the previous reviewer, the article would be significantly strengthened by a discussion of Tsai and colleagues’ recommendations for analytical approaches and a true discussion of the types of additive or interactive evaluations in the studies listed.

- Tsai, A. C., & Burns, B. F. (2015). Syndemics of psychosocial problems and HIV risk: A systematic review of empirical tests of the disease interaction concept. *Social Science & Medicine*, *139*, 26-35. ^1^
- Stall, R., Coulter, R. W., Friedman, M. R., & Plankey, M. W. (2015). Commentary on “Syndemics of psychosocial problems and HIV risk: A systematic review of empirical tests of the disease interaction concept” by A. Tsai and B. Burns. ^2^
- Tsai, A. C., & Venkataramani, A. S. (2016). Syndemics and health disparities: a methodological note. *AIDS and Behavior*, *20*(2), 423-430. ^3^
- Tsai, A. C. (2018). Syndemics: a theory in search of data or data in search of a theory?. *Social Science & Medicine*, *206*, 117-122. ^4^
- Mendenhall, E., Newfield, T., & Tsai, A. C. (2021). Syndemic theory, methods, and data. *Social science & medicine (1982)*. ^5^

The inclusion of academic institutions, authors, and countries in the assessment is lacking recommendations. What are the authors hoping readers do with this information? How does it facilitate or foster collaboration? How would the authors recommend better support of syndemic research in low- and middle-income countries? At present, this information does not add additional value to the study.

Is the work clearly and accurately presented and does it cite the current literature?

Partly

If applicable, is the statistical analysis and its interpretation appropriate?

I cannot comment. A qualified statistician is required.

Are all the source data underlying the results available to ensure full reproducibility?

Yes

Is the study design appropriate and is the work technically sound?

Yes

Are the conclusions drawn adequately supported by the results?

Partly

Are sufficient details of methods and analysis provided to allow replication by others?

Yes

Reviewer Expertise:

I am a reproductive epidemiologist and have published research examining social determinants of sexually transmitted infections, some which apply a syndemic framework.

I confirm that I have read this submission and believe that I have an appropriate level of expertise to confirm that it is of an acceptable scientific standard, however I have significant reservations, as outlined above.

## References

[1] : Syndemics of psychosocial problems and HIV risk: A systematic review of empirical tests of the disease interaction concept. *Soc Sci Med* .2015;139: 10.1016/j.socscimed.2015.06.024 26-35 10.1016/j.socscimed.2015.06.024 26150065PMC4519429

[2] : Commentary on "Syndemics of psychosocial problems and HIV risk: A systematic review of empirical tests of the disease interaction concept" by A. Tsai and B. Burns. *Soc Sci Med* .2015;145: 10.1016/j.socscimed.2015.07.016 129-31 10.1016/j.socscimed.2015.07.016 26254086

[3] : Syndemics and Health Disparities: A Methodological Note. *AIDS Behav* .2016;20(2) : 10.1007/s10461-015-1260-2 423-30 10.1007/s10461-015-1260-2 26662266PMC4755906

[4] : Syndemics: A theory in search of data or data in search of a theory?. *Soc Sci Med* .206: 10.1016/j.socscimed.2018.03.040 117-122 10.1016/j.socscimed.2018.03.040 29628175PMC6613368

[5] : Syndemic theory, methods, and data. *Social Science & Medicine* .2022;295: 10.1016/j.socscimed.2021.114656 10.1016/j.socscimed.2021.114656 PMC866995034949486

# Response

**Published:** 2023-01-25

**Authors:** Samia Tasnim

**Affiliations:** Texass A&M School of Public Health, USA

**Comment:**

 The authors of the present article provided a robust review of the literature examining “Syndemics” in multiple areas of emphasis and with various health indicators and outcomes. This article attempts to provide an overview of syndemic named literature published in the past 20 years and highlights both the researchers, institutions, and countries within which this work is being conducted.

 An understanding of the scope of syndemic work being conducted globally is helpful, however, the article lacks any examination of the statistical approaches used across the articles included and overlooks the relatively robust discourse in the literature on criticisms of commonly used analytical approaches to syndemic research. As mentioned by the previous reviewer, the article would be significantly strengthened by a discussion of Tsai and colleagues’ recommendations for analytical approaches and a true discussion of the types of additive or interactive evaluations in the studies listed.

- Tsai, A. C., & Burns, B. F. (2015). Syndemics of psychosocial problems and HIV risk: A systematic review of empirical tests of the disease interaction concept. *Social Science & Medicine*, *139*, 26-35. 1
- Stall, R., Coulter, R. W., Friedman, M. R., & Plankey, M. W. (2015). Commentary on “Syndemics of psychosocial problems and HIV risk: A systematic review of empirical tests of the disease interaction concept” by A. Tsai and B. Burns. 2

Tsai, A. C., & Venkataramani, A. S. (2016). Syndemics and health disparities: a methodological note. *AIDS and Behavior*, *20*(2), 423-430. 3

- Tsai, A. C. (2018). Syndemics: a theory in search of data or data in search of a theory?. *Social Science & Medicine*, *206*, 117-122. 4
- Mendenhall, E., Newfield, T., & Tsai, A. C. (2021). Syndemic theory, methods, and data. *Social science & medicine (1982)*. 5

**Response:**

 The goal of a scientometric study is to examine the meta-knowledge development in a given topic in terms of research growth and trends and in this study we evaluated the scientific development of syndemics and related health and social issues. We have reviewed the methodology of the studies identified including the statistical approaches. Critical evaluation of the approaches was not possible as it was out of the scope of the present study.

 “It is also crucial to highlight the existing debate within the field of syndemics is the framing and the statistical approaches used to establish relationships between the diseases and outcomes. Considering the complex multilevel involvement of syndemics Tsai et al and colleagues believe that most empirical studies purporting to validate the theory lack in statistical robustness. Tsai identified that most studies in the field of syndemics demonstrate a statistically significant association between psychosocial problems and health outcomes using simple linear models, very few studies assess the how these psychosocial problems interact to magnify the outcomes as they only use summative approach for measuring the effect. Additionally, the summative approach is only successful to measure the changes when the effect sizes are small. Authors suggested to use the summative model using the count variable approach to conduct a latent factor analysis for confirming that a single parameter model is adequate. They emphasized on sharpening the theory’s prediction approaches by incorporating advanced systems level approaches.

 Stall  argued in favor of the summative approach saying that majority of the existing data in this field comes from self-reported data from cross-sectional studies. Applying more rigorous data analysis method would require long-term cohort studies designed to establish the theory. However, considering the current nature of the fundings, implementing such resource intensive cohort study is not feasible. Due to the physical and psychological burden of these diseases (especially HIV) on highly vulnerable marginalized communities such as young gay black men or transgender women, the current approach is acceptable as it can demonstrate the dangerous existence of these co-morbidities highlighting the needs for prevention efforts from practitioners and policy makers.”

**Comment:**

 The inclusion of academic institutions, authors, and countries in the assessment is lacking recommendations. What are the authors hoping readers do with this information? How does it facilitate or foster collaboration? How would the authors recommend better support of syndemic research in low- and middle-income countries? At present, this information does not add additional value to the study.

**Response:**

 Thank you for your insightful comment regarding the recommendation about research institutions, authors, and countries. We have added additional recommendations based on our findings in the discussion in the revised manuscript. 

 “It is critical to foster collaborative efforts among researchers across the globe to expand the ongoing research in thef1000 field of syndemics by including diverse perspectives. The present study has highlighted which institutions, mainly from high-income countries, have expertise in syndemics research. An ideal next step would be to focus on developing long-term collaboration by resource sharing and capacity building with scholars from LMICs to amplify their work. International public health organizations can establish global strategic planning groups that help fund collaborative work to address syndemics across nations. These groups could also concentrate on implementing multilevel strategies involving researchers, policymakers, health workers, and other key stakeholders to achieve a sustainable solution.”

# Peer review: Reviewer response for version 1

**DOI:** 10.5256/f1000research.77914.r125796

**Published:** 2022-03-11

**Authors:** Nicola Bulled

**Affiliations:** 1 InCHIP, University of Connecticut, Storrs, CT, USA; 2 School of Public Health, Brown University, Providence, RI, USA

The article, “Global research on syndemics: a meta-knowledge analysis (2001-2020)” for consideration in F1000 Research, offers a detailed meta-knowledge analysis of published syndemics literature from 2001-2020. Having conducted a review of syndemics literature myself, I agree with the approach taken by the authors regarding search engine, and generally agree with the analysis offered by the data generated. I do, however, feel the authors need to offer a more critical assessment of the analysis regarding what it reveals about the application of the theory, drawing on current conversations.

 A couple of points of clarification are needed regarding how the data is analyzed. What is the relevance of bibliographic elements with reference to how the work is related? It is unclear to me how these elements are included or not in a network analysis of similarities. Incorporate the dates of the other reviews conducted. The reviews mentioned included fewer publications because their dates and in some cases topics were significantly narrower. Also include additional reviews that have not been mentioned, including that by Tsai and Burns 2015.

 I encourage the authors to highlight the limitations of the approach at the outset of the methods rather than in a limitations section at the end. One of the primary limitations is that the authors do not identify what of the literature calling itself “syndemics” is actually a syndemic analysis. In one of the prior reviews of syndemic literature conducted (mentioned in this manuscript), only 12% of the literature reviewed actually met a syndemics definition. If that is the case in your analysis, then close to 90% of the literature included in this analysis is likely not syndemics at all. This needs to be considered as a limitation at the outset of the study. Or at least, the reader needs to be made aware of this problem. The discussion of the prior review did not lay this out clearly enough.

 This is my primary concern for this study. While the results are very interesting, I feel that the story the results tell are not presented in the context of current syndemic theory conversations. The authors fail to include any of the work of A. Tsai, who has strongly critiqued syndemic theory as lacking evidence. A special issue of Social Science and Medicine published between 2019-2022 includes different work attempting to evidence syndemics. Many of these publications do not. What has been recognized is that the method to assess a syndemic arrangement introduced by R. Stall in 2005 actually is a summative score, with more “syndemic” factors present resulting in worse outcomes. This does not indicate a syndemic arrangement with synergy occurring between the factors. Stall himself has agreed that his analytical approach was wrong. The data presented in this study indicates the significant impact this work of Stall has had in directing syndemic literature, completely changing the trajectory of the theory and the approaches to assessing the theory. Stall is one of the top 10 authors and, as shown in Figure 7, has significantly impacted other key authors in the field. Many of the examples offered on pages 12-13 fall into this erroneous application of syndemics. While this may be where syndemics literature is at present, the authors should indicate the problem. Scholars appear to be using “syndemics” as a fashionable new term to describe co-morbidities, with some acknowledging social elements, but many not offering much at all, and few considering evaluating synergistic rather than summative relationships.

 The assessment of institutions also does not offer a great deal. I don’t understand the network dynamic shown, how are these relationships determined? The statistics of frequency per institution need to be proportional to the number of scholars present in the institution or at least offer some indication of size. In this light, UCONN might be producing significantly more than other institutions, which would make sense given as that is where Merrill Singer is based. The authors offer no assessment of why these arrangements are the way they are. In which case, it isn’t clear what the value of the data is. Similarly, the authors do not interrogate the bibliographic data presented. While it is interesting to consider number of authors, number of documents per author, and number of keywords. What does this really tell us? If the network is generated on these similarities, I don’t see the value of the network.

 One final point of note, in the top 10 articles listed, the Lancet piece on global obesity syndemic is second. This proved a highly contentious article in the field of syndemics researchers as it posited that a global syndemic could exist. For syndemics scholars that recognize the influence of social factors, this was not possible. An obesity epidemic in India is not socially the same as an obesity epidemic in the US or South Africa or Australia. What this study did, much like Stall’s, was set syndemics theory on another course of inaccurate application.

**The authors need to:**

- Include the work of Tsai and the conversations he has provoked regarding the direction of syndemics' work.
- Interrogate the data presented in reference to Tsai’s work, where has syndemic theory gone, and is this problematic.
- Don’t use examples of syndemics literature that isn’t presenting a syndemics arrangement, unless the aim is to indicate how it isn’t a syndemic arrangement (references such as: 22, 29, 30, 31, 32, 33, 34, 35, 36, 37). Anything that indicates that more factors results in worse outcomes is using an additive/summative approach and not assessing for synergy between factors.
- Fix the typo “bib-bio”, it comes up twice.
- Offer a richer analysis of the data presented, including discussing the relevance of bibliographic elements, presenting institutional publication records relative to institutional size, and assessing how different frameworks of understanding syndemic theory and evaluating syndemics influenced the literature.

Is the work clearly and accurately presented and does it cite the current literature?

Partly

If applicable, is the statistical analysis and its interpretation appropriate?

Yes

Are all the source data underlying the results available to ensure full reproducibility?

Yes

Is the study design appropriate and is the work technically sound?

Yes

Are the conclusions drawn adequately supported by the results?

Partly

Are sufficient details of methods and analysis provided to allow replication by others?

Yes

Reviewer Expertise:

I am a syndemics researcher having published numerous syndemics papers with Merrill Singer, including the heavily cited Lancet piece noted in this manuscript as the 6th most cited syndemics article. I have worked on syndemics for close to 15 years. My primary focus area is infectious disease and syndemic theory development, including the evidencing of the theory.

I confirm that I have read this submission and believe that I have an appropriate level of expertise to confirm that it is of an acceptable scientific standard, however I have significant reservations, as outlined above.

# Response

**Published:** 2023-01-25

**Authors:** Samia Tasnim

**Affiliations:** Texass A&M School of Public Health, USA

**Comment:**

 The article, “Global research on syndemics: a meta-knowledge analysis (2001-2020)” for consideration in F1000 research, offers a detailed meta-knowledge analysis of published syndemics literature from 2001-2020. Having conducted a review of syndemics literature myself, I agree with the approach taken by the authors regarding search engines and generally agree with the analysis offered by the data generated. I do, however, feel the authors need to offer a more critical assessment of the analysis regarding what it reveals about the application of the theory, drawing on current conversations.

**Response:**

 We appreciate the valuable comments of the respected reviewer. The goal of a scientometric study is to examine the meta-knowledge development in a given topic in terms of research growth and trends ^1-3^. It is used for unraveling and mapping the cumulative scientific knowledge in well-established fields by objective analysis of large volumes of unstructured data. It aims to uncover emerging trends in the field by highlighting the article and journal performances, collaboration patterns, research constituents, along with the intellectual structure of a specific domain. In this current study, we evaluated the scientific development of syndemics and related health and social issues. This approach is significantly different than what can be done in a systematic review or a critical review. However, the scope of critical evaluation of the research topics in light of the previous research and academic discourses remain relevant. In this revised version, we revisited those areas and provided updated information and explanations that may address the shared concerns. Reflection of our critical assessment can be found in the discussion section.

**Comment:**

 A couple of points of clarification are needed regarding how the data is analyzed. What is the relevance of bibliographic elements with reference to how the work is related? It is unclear to me how these elements are included or not in a network analysis of similarities.

 Incorporate the dates of the other reviews conducted. The reviews mentioned included fewer publications because their dates and in some cases topics were significantly narrower. Also include additional reviews that have not been mentioned, including that by Tsai and Burns 2015

**Response:**

 As mentioned in the methods section, we have included all the articles meeting the inclusion criteria indexed in Web of Science until 6/26/2022. The article by Tsai et al., might have been missed due to mismatched indexing. We have also mentioned in our limitation that due to lack of universal indexing of scientific journals there is a possibility of missing articles not indexed in this major database.

 We have also reviewed the analyzed works and relevant literature as suggested. We found that some of the studies indexed outside of WoS were not included in the current study. However, adding them to the dataset from non-homogenous bibliographic sources would create further discrepancies on bibliometric indicators. Therefore, we confined our work within the scope of WoS collection and discussed this elaborately both in the methods and discussion sections of our manuscript. The rest of the network analyzes were revisited and they are consistent with the WoS collection on syndemics, which we believe cover the majority of, if not all, scientific publications on this topic.

**Comment:**

 I encourage the authors to highlight the limitations of the approach at the outset of the methods rather than in a limitations section at the end. One of the primary limitations is that the authors do not identify what of the literature calling itself “syndemics” is actually a syndemic analysis. In one of the prior reviews of syndemic literature conducted (mentioned in this manuscript), only 12% of the literature reviewed actually met a syndemics definition. If that is the case in your analysis, then close to 90% of the literature included in this analysis is likely not syndemics at all. This needs to be considered as a limitation at the outset of the study. Or at least, the reader needs to be made aware of this problem. The discussion of the prior review did not lay this out clearly enough

**Response:**

 Thank you for mentioning this critical problem. We acknowledge this issue and we have revised the methods and limitations accordingly to make the readers aware of this problem. 

 “Due to discrepancy in the framing of syndemics in the published literature, the coexistence of multiple health problems without specifying the underlying bio-bio or bio-social relationships is often studied within the syndemic framework. ^9^ Thus studies titled syndemics can be included in the analysis despite the differences in the scope of their work. This issue has been elaborated in the limitations section.”

**Comment:**

 This is my primary concern for this study. While the results are very interesting, I feel that the story the results tell are not presented in the context of current syndemic theory conversations. The authors fail to include any of the work of A. Tsai, who has strongly critiqued syndemic theory as lacking evidence. A special issue of Social Science and Medicine published between 2019-2022 includes different work attempting to evidence syndemics. Many of these publications do not. What has been recognized is that the method to assess a syndemic arrangement introduced by R. Stall in 2005 actually is a summative score, with more “syndemic” factors present resulting in worse outcomes. This does not indicate a syndemic arrangement with synergy occurring between the factors. Stall himself has agreed that his analytical approach was wrong. The data presented in this study indicates the significant impact this work of Stall has had in directing syndemic literature, completely changing the trajectory of the theory and the approaches to assessing the theory. Stall is one of the top 10 authors and, as shown in Figure 7, has significantly impacted other key authors in the field. Many of the examples offered on pages 12-13 fall into this erroneous application of syndemics.While this may be where syndemics literature is at present, the authors should indicate the problem. Scholars appear to be using “syndemics”as a fashionable new term to describe co-morbidities, with some acknowledging social elements, but many not offering much at all, and few considering evaluating synergistic rather than summative relationships.

**Response:**

 We revisited our analyses and also retrieved the data again to cross-check if there was any discrepancies. We included all articles in Web of Science until 6/26/2022 and realized that a major proportion of Tsai’s work was published in 2021-22, whereas our primary analyses included studies up to 2020. By that time, Tsai had 7 publications on syndemics, whereas the last author mentioned in our list had 9 publications. This is the reason why we couldn’t mention Tsai from an objective viewpoint. Perhaps this is also the reason why our paper may not inform anything regarding articles that were published beyond the scope of our dataset.

 As we explained in the methods section of our revised submission, we included any work related to syndemics irrespective of the definitions or frameworks used by the respective authors. We welcome the discussion on the references related to Stall and colleagues. We have presented arguments from both sides in the discussion to inform the readers. We have also mentioned the differences in the opinions and objectively reflected its impact on research synthesis in the discussion section of this revised submission.

 “It is also crucial to highlight the existing debate within the field of syndemics is the framing and the statistical approaches used to establish relationships between the diseases and outcomes. Considering the complex multilevel involvement of syndemics Tsai et al and colleagues believe that most empirical studies purporting to validate the theory lack in statistical robustness. Tsai identified that most studies in the field of syndemics demonstrate a statistically significant association between psychosocial problems and health outcomes using simple linear models, very few studies assess the how these psychosocial problems interact to magnify the outcomes as they only use summative approach for measuring the effect. Additionally, the summative approach is only successful to measure the changes when the effect sizes are small. Authors suggested to use the summative model using the count variable approach to conduct a latent factor analysis for confirming that a single parameter model is adequate. They emphasized on sharpening the theory’s prediction approaches by incorporating advanced systems level approaches.

 Stall argued in favor of the summative approach saying that majority of the existing data in this field comes from self-reported data from cross-sectional studies. Applying more rigorous data analysis method would require redesigning studies such as long-term cohorts to collect data to establish the theory. However, considering the current nature of the funding, implementing such resource intensive cohort study is not feasible. Due to the physical and psychological burden of these diseases (especially HIV) on highly vulnerable marginalized communities such as young gay black men or transgender women, the current approach is acceptable as it can demonstrate the dangerous existence of these co-morbidities highlighting the needs for prevention efforts from practitioners and policy makers.”

**Comment:**

 The assessment of institutions also does not offer a great deal. I don’t understand the network dynamic shown, how are these relationships determined? The statistics of frequency per institution need to be proportional to the number of scholars present in the institution or at least offer some indication of size. In this light, UCONN might be producing significantly more than other institutions, which would make sense given as that is where Merrill Singer is based. The authors offer no assessment of why these arrangements are the way they are. In which case, it isn’t clear what the value of the data is. Similarly, the authors do not interrogate the bibliographic data presented. While it is interesting to consider number of authors, number of documents per author, and number of keywords. What does this really tell us? If the network is generated on these similarities, I don’t see the value of the network.

**Response:**

 As mentioned in the method section of the paper we conducted descriptive analysis of key bibliometric characteristics, institutions conducting research are commonly considered as a key characteristics in the bibliometric analysis as it can provide ideas about distribution of resources and potential areas for collaboration between institutions across the globe ^4-6^. However, it was not possible to further the analysis at individual researcher level as this information was not included in the bibliometric data retrieved from the database. Moreover, there is a possibility for the researchers to change their work affiliation or hold more than one affiliation, thus analysis based on publications as proportional to the number of scholars will vary but publications from institutions will remain relatively constant. Our approach is consistent with previous bibliometric studies that offered aggregated outcomes for authors and institutions ^7,8^. Lastly, the value of the network, particularly those illustrating clusters of research nodes, are useful to visualize research hotspots and themes. As noted in earlier works ^9,10^ those insights not only inform current research trends, but also provide critical insights on research gaps in a scientific area. Both in our results and discussion, we discussed those hotspots and potential gaps in syndemic literature, which can be considered as the added contributions of this study.

 References:

1. Abdalla SM, Solomon H, Trinquart L, Galea S. What is considered as global health scholarship? A meta-knowledge analysis of global health journals and definitions. BMJ global health. 2020 Oct 1;5(10):e002884.
2. Wallin JA. Bibliometric methods: pitfalls and possibilities. Basic & clinical pharmacology & toxicology. 2005 Nov;97(5):261-75.
3. Chen C, Song M. Visualizing a field of research: A methodology of systematic scientometric reviews. PloS one. 2019 Oct 31;14(10):e0223994.
4. Sweileh, W. M. (2022). Bibliometric analysis of scientific research publications on COVID-19 vaccine hesitancy (2020–2021).
5. Sweileh, W. M. (2020). Bibliometric analysis of global scientific literature on vaccine hesitancy in peer-reviewed journals (1990–2019). *BMC public health*, *20*(1), 1-15.
6. Song, P., & Wang, X. (2020). A bibliometric analysis of worldwide educational artificial intelligence research development in recent twenty years. *Asia Pacific Education Review*, *21*(3), 473-486.
7. Mooghali A, Alijani R, Karami N, Khasseh AA. Scientometric analysis of the scientometric literature. International Journal of Information Science and Management (IJISM). 2012 Jul 18;9(1):19-31.
8. Ramy A, Floody J, Ragab MA, Arisha A. A scientometric analysis of Knowledge Management Research and Practice literature: 2003–2015. Knowledge Management Research & Practice. 2018 Jan 2;16(1):66-77.
9. Chen C, Dubin R, Kim MC. Emerging trends and new developments in regenerative medicine: a scientometric update (2000–2014). Expert opinion on biological therapy. 2014 Sep 1;14(9):1295-317.
10.  Zhao X. A scientometric review of global BIM research: Analysis and visualization. Automation in Construction. 2017 Aug 1;80:37-47.
